# Synergistic Interactions between Alzheimer’s Aβ40 and Aβ42 on the Surface of Primary Neurons Revealed by Single Molecule Microscopy

**DOI:** 10.1371/journal.pone.0082139

**Published:** 2013-12-02

**Authors:** Chun-Chieh Chang, John Christian Althaus, Cynthia J. L. Carruthers, Michael A. Sutton, Duncan G. Steel, Ari Gafni

**Affiliations:** 1 Department of Biophysics, University of Michigan, Ann Arbor, Michigan, United States of America; 2 Molecular and Behavioral Neuroscience Institute, University of Michigan, Ann Arbor, Michigan, United States of America; 3 Department of Molecular and Integrative Physiology, University of Michigan, Ann Arbor, Michigan, United States of America; 4 Department of Physics, University of Michigan, Ann Arbor, Michigan, United States of America; 5 Department of Electrical Engineering and Computer Science, University of Michigan, Ann Arbor, Michigan, United States of America; 6 Department of Biological Chemistry, University of Michigan, Ann Arbor, Michigan, United States of America; Louisiana State University Health Sciences Center, United States of America

## Abstract

Two amyloid-β peptides (Aβ40 and Aβ42) feature prominently in the extracellular brain deposits associated with Alzheimer’s disease. While Aβ40 is the prevalent form in the cerebrospinal fluid, the fraction of Aβ42 increases in the amyloid deposits over the course of disease development. The low *in vivo* concentration (pM-nM) and metastable nature of Aβ oligomers have made identification of their size, composition, cellular binding sites and mechanism of action challenging and elusive. Furthermore, recent studies have suggested that synergistic effects between Aβ40 and Aβ42 alter both the formation and stability of various peptide oligomers as well as their cytotoxicity. These studies often utilized Aβ oligomers that were prepared in solution and at μM peptide concentrations. The current work was performed using physiological Aβ concentrations and single-molecule microscopy to follow peptide binding and association on primary cultured neurons. When the cells were exposed to a 1:1 mixture of nM Aβ40:Aβ42, significantly larger membrane-bound oligomers developed compared to those formed from either peptide alone. Fluorescence resonance energy transfer experiments at the single molecule level reveal that these larger oligomers contained both Aβ40 and Aβ42, but that the growth of these oligomers was predominantly by addition of Aβ42. Both pure peptides form very few oligomers larger than dimers, but either membrane bound Aβ40/42 complex, or Aβ40, bind Aβ42 to form increasingly larger oligomers. These findings may explain how Aβ42-dominant oligomers, suspected of being more cytotoxic, develop on the neuronal membrane under physiological conditions.

## Introduction

Amyloid deposits in the brain, a hallmark of Alzheimer’s disease, are largely composed of two peptides, Aβ40 and Aβ42, produced by the proteolytic cleavage of the amyloid precursor protein by β- and γ-secretases. The two amyloid peptides differ only in the presence of an Ile–Ala dipeptide at the C-terminus of Aβ42 [1]. Although Aβ40 is produced in large abundance over Aβ42, the latter is the majority peptide in the brain plaques [2–4] and an increase in the Aβ42/Aβ40 ratio in the plasma is associated with familial forms of Alzheimer’s disease (AD) [5,6], while a reduction of Aβ42 concentration correlates with a decreased risk for AD [7]. 

Initial reports of a correlation between extent amyloid deposits and AD severity lead to the amyloid hypothesis for the disease; however, extensive recent work has revealed that neuronal damage is associated with small oligomeric species of Aβ, leading to the peptide oligomer hypothesis for AD [8–14]. Indeed, elevated levels of Aβ oligomers have been found to be a better indicator of disease in humans than plaque load [8,9,15,16]. More specifically, stabilized versions of Aβ dimers or dimer aggregates were shown to reduce long-term potentiation in cultured neurons [17,18] and a comparison of cross-linked Aβ dimers, trimers, and tetramers revealed that neurotoxicity increases nonlinearly with oligomer size [19]. Interestingly, numerous studies have shown that when Aβ42 is incubated in solution at µM concentrations it possesses a considerably higher fibril formation rate than Aβ40 [20,21] and also forms larger, more cytotoxic, oligomers [22–24]. 

Two different explanations could be proposed for the observed increase in the risk for AD with increased relative production of Aβ42/Aβ40 ratio: 1. More potent cell disrupting oligomers are formed by Aβ42, and their production rate rapidly increases at higher concentration of this peptide; or 2. Synergistic interactions between Aβ40 and Aβ42 lead to the formation of mixed oligomers that are more pathogenic than oligomers formed by either peptide alone, and whose levels increase with increasing Aβ42 levels. While evidence for Aβ40-Aβ42 interactions has been documented in several of the studies listed above, these were all done using high (µM) concentrations of peptide, at least 1,000 times higher than the pM-nM concentration of Aβ in brain tissue [8,9]. Moreover, the protocols used in these studies employed oligomers that were pre-made in solution and later added to the cultured cells. These oligomers are potentially significantly different, both in composition and properties, from the ones created at physiological peptide concentrations and in association with the neuronal membrane. 

Studies using physiological concentrations of peptide, however, are experimentally challenging since they require cell–bound Aβ species to be individually monitored over long periods of time at nanomolar to picomolar levels, the cell disrupting ones (likely a minority) need to be identified and their size, peptide composition and cellular interactions need to be characterized. An added difficulty is the fact that cell-bound Aβ oligomers are heterogeneous and metastable, continuously interconverting between species [25–27] and their cellular membrane interactions are potentially complex and variable. For example, there is evidence that the peptide may associate with specific cellular receptors or protein complexes (e.g. NMDA receptors [28,29] and α7 nicotinic acetylcholine receptors [30]), it may associate with phosphatidylserine in the membrane [31], or bind to and insert directly into the lipid bilayer [32,33]. Since Aβ is an amphiphilic peptide, the initial binding affinity of Aβ is correlated with the polar interactions and anionic charge of lipid head groups [34]. Each of these modes of interaction reflects a different potential pathway to cell disruption. 

Recently, our group [35–38] and others [39,40] employed single molecule microscopy, both in Total Internal Reflection Fluorescence (TIRF) and confocal to study the assembly and size distribution of Aβ oligomers generated at physiological concentrations on the surface of model membranes, PC12 cells, SH-SY5Y neuroblastoma cells and cultured neuronal cells. We found that at these low peptide concentrations, only insignificant oligomerization occurred in solution even after five days of incubation, whereas the membrane facilitated the formation of surface bound oligomers with an obviously larger size distribution. Again with single molecule microscopy we also found that Aβ40 oligomers size correlates with conductance changes in a model membrane [35]. 

In the present study we extend our previous work to focus on synergistic interactions between Aβ40 and Aβ42 during their assembly into oligomers on the surface of primary cultured neurons, at physiological concentrations of peptides. As discussed above, such synergy is potentially of great significance for the development of our understanding of the molecular events underlying cell disruption in AD. The identification of those oligomers that develop on the membrane at nM peptide concentrations is of special relevance.

To monitor peptide binding to the neuronal membrane and its assembly into oligomers we applied single-molecule microscopy. The results show that, at nM concentrations, both homogeneous (unmixed) Aβ40 and Aβ42 remained mostly monomeric in solution even after prolonged incubation, and formed primarily dimers on the surface of neurites showing no further growth [38]. In contrast, when the cells were exposed to a 1:1 mixture of Aβ40 and Aβ42, significantly larger membrane-bound oligomers developed. By applying fluorescence resonance energy transfer (FRET) at the single molecule level we document that these larger oligomers contain both Aβ40 and Aβ42 and are stable against dissociation over several days. More strikingly, these studies reveal that the growth of the mixed oligomers occurs exclusively by the addition of Aβ42, such that the membrane bound Aβ42/Aβ40 ratio continuously increases with time.  While it was not practical, with the limited “life expectancy” of cultured neurons, to allow for amyloid fibrils to form, the results provide picture consistent with the ratio of peptides found in the deposits seen in the brain of AD patients and transgenic mice [41–43]. 

Our results reveal that in the formation of oligomers on the neuronal membrane, there is a strong cooperativity between Aβ40 and Aβ42 that results in an increase in the fraction and absolute numbers of oligomers larger than the dominant dimers and trimers formed by either Aβ40 or Aβ42 in isolation. The data further indicates that this shift in the reaction products is due to either membrane bound Aβ40/42 complex, or Aβ40, that seeds the addition of Aβ42 to form increasingly larger oligomers. Potentially of significance to cellular toxicity, the findings reported here may explain how Aβ42-dominant oligomers, suspected of being more cytotoxic, develop on the neuronal membrane under physiological conditions.

## Materials and Methods

### Peptide Preparation

N-terminally HiLyteFluor 555 labeled Aβ40, and N-terminally HiLyteFluor 647 labeled Aβ42 (Aβ40-HL555 and Aβ42-HL647, respectively) were obtained from Anaspec (Freemont, CA). Aβ peptides were dissolved in 1% NH_4_OH at 0.1 mg/mL and vortexed for 30 s. The peptide solutions were aliquoted into individual microtubes, lyophilized and the solids stored at -20°C. To prepare fresh Aβ samples, single aliquots were dissolved in 10 mM sodium phosphate buffer, pH 7.4, to a concentration of 1-2 µM (as determined spectrophotometrically using ε_555_=150,000 and ε_647_=250,000). Freshly prepared Aβ were further diluted down to final concentrations within 15 minutes. 

Numerous control experiments have been done in our laboratory and others suggest that various forms of N-terminally labeled Aβ behave similarly to unlabeled Aβ in terms of fibrilization [44], ability to permeabilize synthetic membranes [27,35] as well as rat basophilic leukemia cell-derived membrane blebs [36], toxicity to cultured cells [45] and microglial activities inside the mouse cortex [46].

### Primary Rat Hippocampal Cell Culture

Dissociated neuron cultures were made from newborn pups (P0−P2). Rats were euthanized by decapitation immediately prior to brain dissection and tissue collection. This procedure was carried out in strict accordance with the recommendations in the Guide for the Care and Use of Laboratory Animals of the National Institutes of Health. The protocol was approved by the University Committee on Use and Care of Animals (UCUCA) at the University of Michigan. Primary rat hippocampal neuron cultures were prepared as described [47]. Cells were plated at 30,000/well on 14 mm poly-D-lysine coated glass coverslips adhered to 35 mm culture dishes (MatTek, Ashland, MA). Imaging experiments were performed between DIV 12 and DIV 18. For single molecule oligomer size measurement experiments, cells were incubated for 10 minutes or 48 hours at 37°C in HBS (HEPES-Buffered Saline: 119 mM NaCl, 5 mM KCl, 2 mM CaCl_2_, 2 mM MgCl_2_, 30 mM Glucose, 10 mM HEPES, pH 7.4) containing 2 nM Aβ40-HL555 or Aβ42-HL647 or 4 nM mixed Aβ40-HL555 and Aβ42-HL647 at 1:1 ratio. Before imaging, cells were washed three times in HBS and then imaged within two hours.

### Fluorescence Lifetime Imaging

Fluorescence lifetime imaging microscopy (FLIM) was performed at the University of Michigan's Single Molecule Analysis in Real-Time (SMART) Center and was measured by time-correlated single-photon counting (TCSPC) by ALBA microscope system (ISS, Champaign, IL). The microscope was Olympus IX-81, equipped with a 37°C temperature controlling stage, a 1.2NA 60X water-immersion objective (Olympus) and imaged by two APDs. The excitation source was Fianium SC 400-6-PP with acousto-optic tunable filters (AOTF). Laser excitation was selected at 532nm and 635nm with power 41.5 μW and 34.6 μW before the objective respectively. The emission filter for AB40-HL555 and AB42-HL647 were 582/75 and 697/75 nm band pass filter (Semrock) respectively. The dichroic mirror was 405/470/532/632 quadband dichroic mirror (Alluxa, Santa Rosa, CA). The lifetime is fitted by VistaVision software (ISS, Champaign, IL) with one exponential decay curve. Detailed FLIM analysis is described in Supporting Information and Figure S1 in File S1.

### Confocal Mode Integrated Intensity-based Oligomer Size Determination

**Figure 1:**
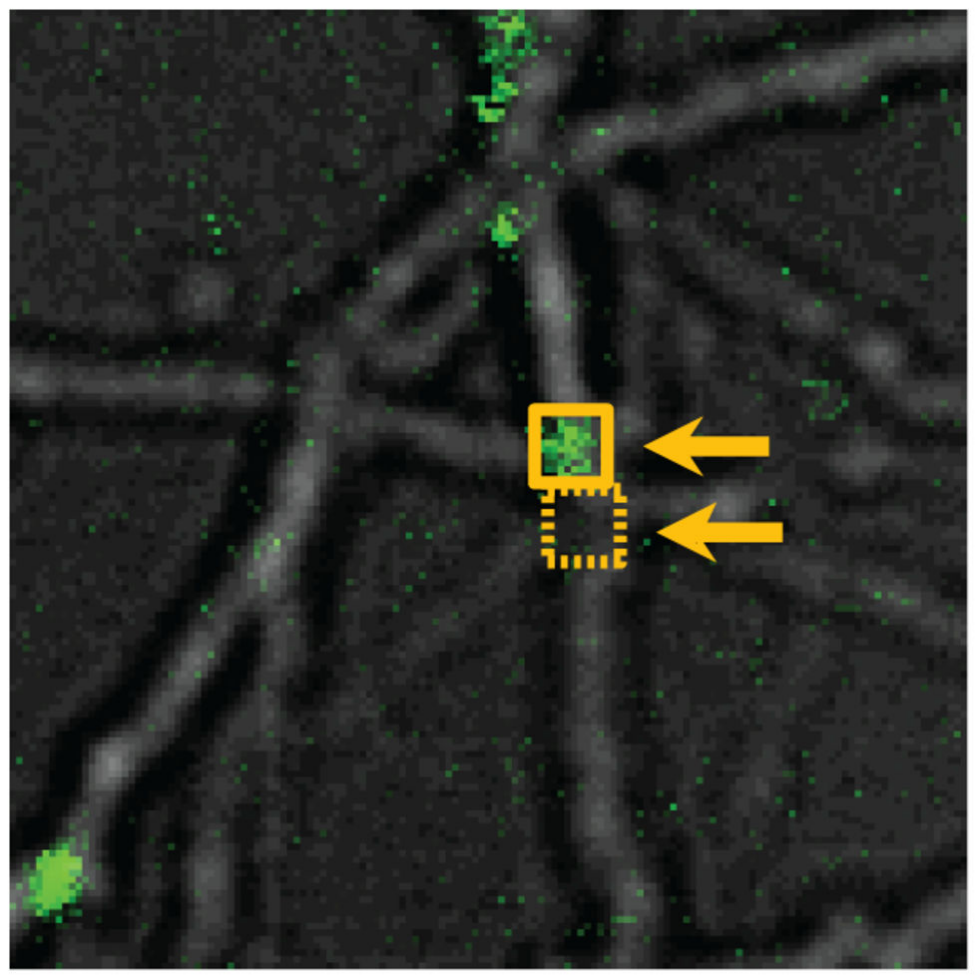
Aβ oligomer size is determined by its fluorescence intensity. The oligomer size were defined by the fluorescence spot whose maxima fell on or within 500 nm of a neurite and were boxed with a 12 pixel x 12 pixel (~1.5 µm x 1.5 µm) region of interest (solid square). This integrated intensity was subtracted by the adjacent background fluorescence counts (dashed square) and then divided by fluorescence intensity per molecule (monomer) to yield the number of oligomer.

We have used confocal mode fluorescence intensity to measure oligomer size on both black lipid membranes and cell membranes [35,36,38]. To measure oligomer size on living cells, a protocol has been developed to correlate particles’ confocal mode fluorescence intensity values with the number of Aβ monomers they contain [36]. When the laser power is below saturation, the total fluorophore emission varies linearly with Aβ concentrations in solution. Therefore, the slope of total intensity from a given volume versus the number of molecules present in the volume yields intensity per molecule. The fluorescence intensity of an oligomer can be divided by this value to yield the number of Aβ monomers present in the oligomer. The cell-bound oligomers were defined as those fluorescence spots whose maxima fell on or within 500 nm of a neurite and were boxed with a 12 pixel x 12 pixel (~1.5 µm x 1.5 µm) region of interest (Figure 1 solid square). Following subtraction of adjacent background fluorescence counts (Figure 1 dashed square), the integrated fluorescence intensity of each region of interest was divided by fluorescence intensity per molecule to determine oligomer size. However, the fluorescence intensity is partially quenched upon binding to the neuronal membrane. For dynamic quenching, the ratio of intensity of the quenched fluorophore (I_q_) to that of the unquenched fluorophore (I_0_) is equal to the ratio of the fluorophore lifetimes (τ_q_ and τ_0_, respectively) under each condition [48]:

$$\frac{I_{q}}{I_{0}}=\frac{\tau_{q}}{\tau_{0}}$$

The averaged fluorescence lifetimes for Aβ40 and Aβ42 in 10 mM sodium phosphate buffer (τ_0_) were 0.75 and 1.56 ns. And the averaged lifetimes of membrane bound Aβ40 and Aβ42 were 0.48 and 1.24 ns. 

## Results

### Aβ40 and Aβ42 Form Mainly Dimers on Neurites and Show Little Growth upon Incubation

**Figure 2:**
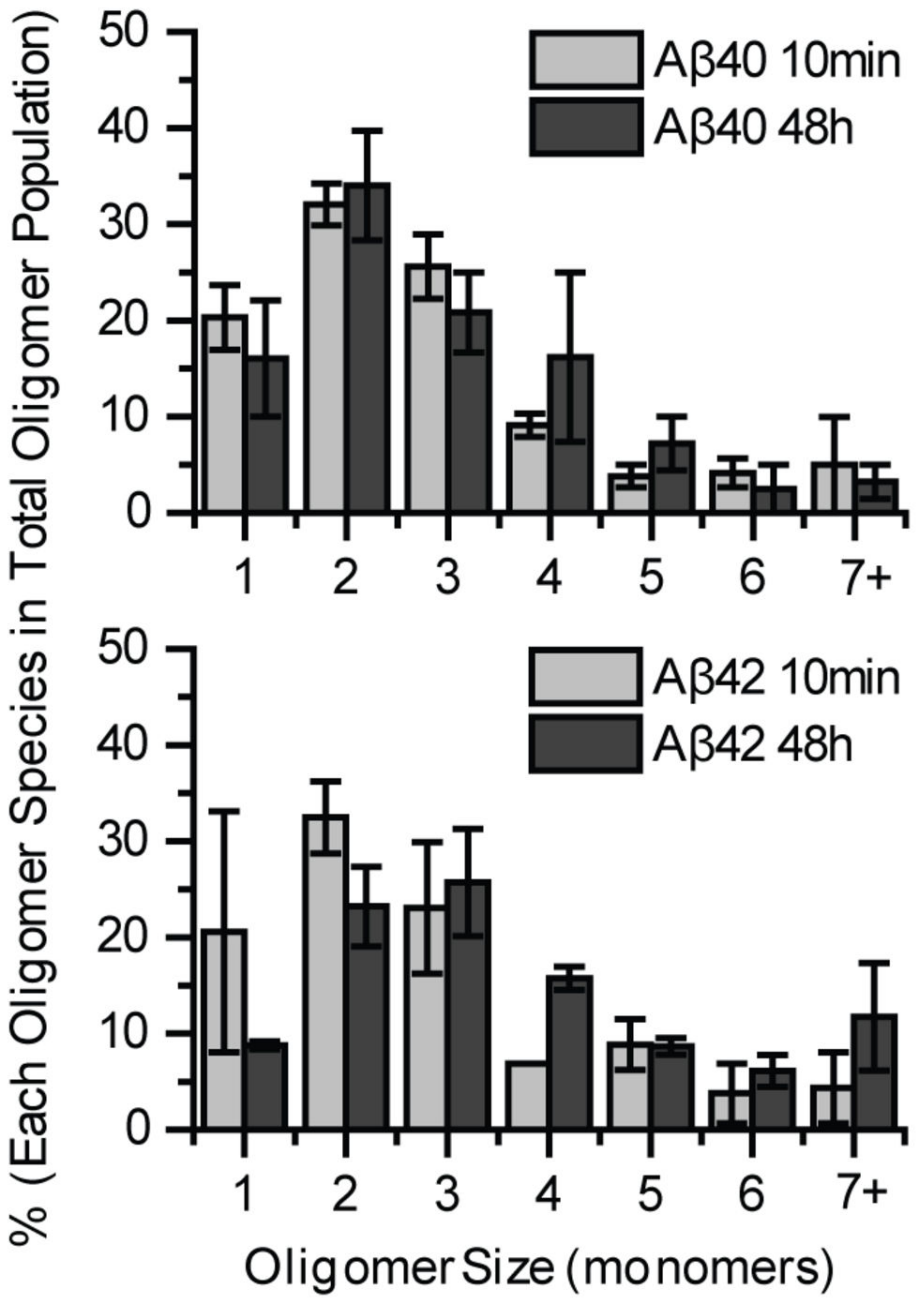
Aβ40 or Aβ42 oligomers form mainly dimers and show little growth on neurites. 2nM Aβ40-HL555 or Aβ42-HL647 was incubated with primary hippocampal neurons for 10 minutes and 48 hours before imaging. Comparison of the oligomeric size distribution between 10 minutes and 48 hours shows limited growth for both Aβ40-HL555 (Mann-Whitney U test, p > 0.1) and Aβ42-HL647 (Mann-Whitney U test, p = 0.001). The distribution is normalized to total Aβ oligomers. Percentages of each condition were calculated from two different experiments, 5 images each. Each image contained at least 50 oligomers. Error bars represent standard deviation of the mean. The percent is obtained by normalizing to the total number of oligomers.

We have previously shown that 1 nM of either HilyteFluor647 labeled Aβ40 (or Aβ42) forms predominantly dimers on neurites, whereas the incubating media still contains 90% monomeric Aβ [38]. This may suggest dimeric Aβ preferentially interacts with the membrane. Similar results have been observed in the current study for using 2 nM Aβ40-HL555 or Aβ42-HL647. Aβ40 species bound to the neuritic membrane are mostly dimeric showing minimal additional growth even after additional 48 hours of incubation. Aβ42 behaved similarly, though some additional growth beyond that at 10 minutes was detected at 48 hours with the appearance of some trimer and larger specie (Figure 2). The incremental growth of Aβ42 (compared to what we reported earlier [38]) could be due to a higher peptide concentration used in the current experiment than the previous work (2 nM vs. 1 nM). Nevertheless, the important point is that the overall oligomeric growth for both Aβ40 and Aβ42 is limited. We note that for the dimers found on the membrane at 10 minutes, we cannot distinguish whether these are formed from bound monomers through assembly on the membrane or from residual dimers in solutions.

### Förster Resonance Energy Transfer (FRET) Confirms Aβ40 and Aβ42 Form Heterogeneous Species on Neurites

**Figure 3:**
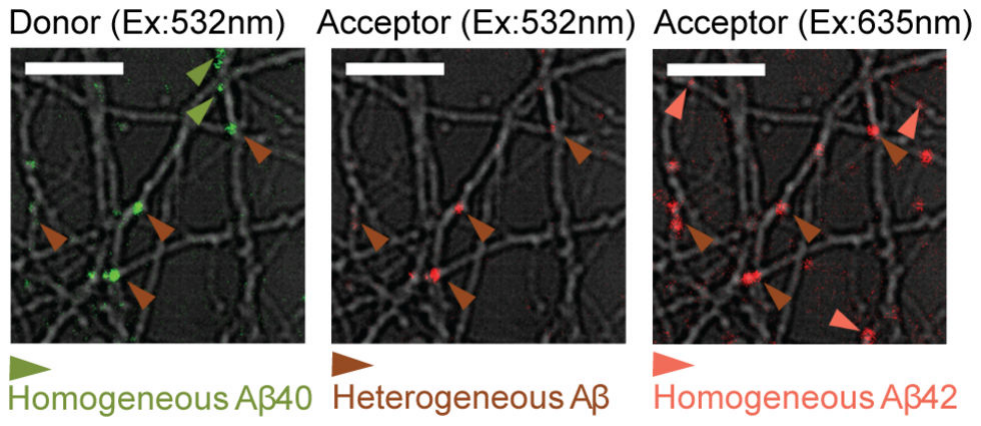
Mixed Aβ40-HL555 and Aβ42-HL647 are incubated with neurons and show FRET. Homogeneous Aβ40 (green arrow) is imaged by 532 nm excitation but does not show signal in acceptor’s channel. Heterogeneous Aβ (brown arrow) is determined by FRET in Acceptor’s channel upon 532 nm excitation. Homogeneous Aβ42 (pink arrow) is imaged by 635 nm excitation but without FRET. Scale bars are 10 µm.

In order to explore potential cooperative interactions between Aβ40 and Aβ42 when they are incubated together with cultured neurons, we employed a FRET pair that enables us to distinguish the heterogeneous oligomers from homogeneous oligomers; only when Aβ42 binds to Aβ40 and forms heterogeneous oligomer do the systems generate a FRET signal (Figure 3). In addition, labeling Aβ40 and Aβ42 with fluorophores that emit significantly different wavelengths enables us to distinguish Aβ40’s signal from Aβ42 when both are present. Aβ40 is labeled with HilyteFluor555, a Cy3 derivative as the FRET donor, and Aβ42 is labeled with HilyteFluor647, a Cy5 derivative as the FRET acceptor. The Förster radius (R_0_) for this pair is estimated ~ 53 Å [49], providing a sensitive reporter on the distance between these two lumiphores. Detailed FRET analysis has been described in Supporting Information and Figure S2 in File S1.

### Number of Heterogeneous Species (i.e., oligomers comprised of both Aβ40 and 42) Increases Over Time due to Continuous Binding of Aβ42 to Heterogeneous Oligomers on the Neurites

**Figure 4:**
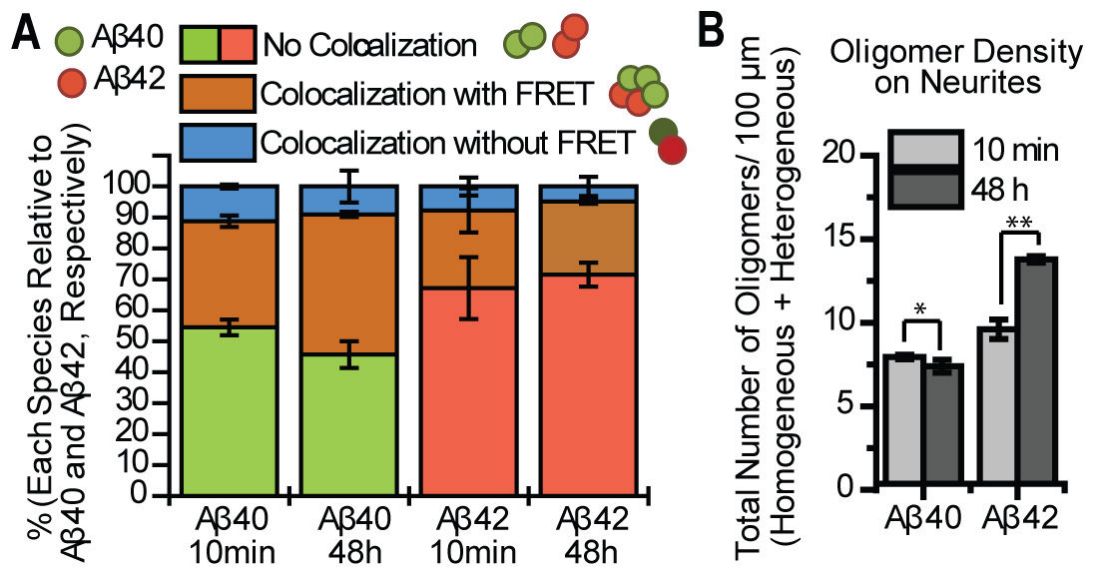
Heterogeneous species increases over time due to continuous binding of Aβ42 to the neurites. 2 nM Aβ40 and 2 nM Aβ42 were mixed and incubated with cultured neurons at the same time. By comparing the population changes of each species, we get general idea of how these species interact over time. (A) The relative number of oligomers of each species in each sample (percentage of each species). The blue shading (colocalization without FRET) represents the co-localized Aβ40 and Aβ42 that do not show a FRET signal. This species accounts for at most 10% for both Aβ40 and Aβ42. The green shaded sections represent the percentage of homogeneous Aβ40 in total Aβ40 species, and show the fraction of homogeneous Aβ40 to decrease over time. The red bar represents the percentage of homogeneous Aβ42 in total Aβ42 species. This number remains almost unchanged over time, indicating the fraction of homogeneous Aβ42 remains unchanged. The brown bar represents the percentage of heterogeneous mixed species in total Aβ40 (left two) or Aβ42 (right two) species. The fraction of heterogeneous species among the whole Aβ40 species increases over time (from 35% to 45%), whereas the fraction of heterogeneous species among whole Aβ42 species remains similar. (B) The density (number of Aβ42 per 100 µm) of Aβ42 oligomers on the neurites (including both homogeneous and heterogeneous species) is slightly higher than Aβ40 at 10 minutes and becomes significantly larger by 48 hours, whereas the total number of Aβ40 is only slightly changed (unpaired two-tailed t-test, *P > 0.1 and **P < 0.05). Data was averaged from two different experiments, at least 5 images each and each image contained at least 50 oligomers. Error bars represent standard deviation of the mean. Figure 5 provides a pictorial display of the implications.

As illustrated in Figure 4, when a mixture of 2 nM Aβ40 and 2 nM Aβ42 is incubated with cultured neurons at the same time, four fluorescent species are detected: homogeneous Aβ40 oligomer (green), homogeneous Aβ42 oligomer (red), heterogeneous Aβ40-Aβ42 oligomer showing FRET (brown), and co-localized Aβ40-Aβ42 oligomers with no FRET (blue). To get information on the changes of Aβ40 and Aβ42’s populations over time, we compared the percentage of each species at two incubation time points. By counting the number of different oligomer species on the neurite, we found that less than 10% of either Aβ40 or Aβ42 oligomers co-localized without showing a FRET signal and that most of the co-localized assemblies containing 40 and 42 showed FRET. Approximately 35% of Aβ40-HL555 oligomers formed FRET pairs with Aβ42-HL647 after 10 minute incubation, and this number increased to ~45% by 48 hours (Figure 4A). This was not due to dissociation of homogeneous Aβ40 oligomers because the total number of Aβ40 oligomers remained unchanged while the total number of Aβ42 oligomers slightly increased over time (Figure 4B). Therefore, the increased fraction of Aβ40 oligomer that is bound to Aβ42 was due to continued binding of solution Aβ42 to homogeneous Aβ40 oligomers over time. 

**Figure 5:**
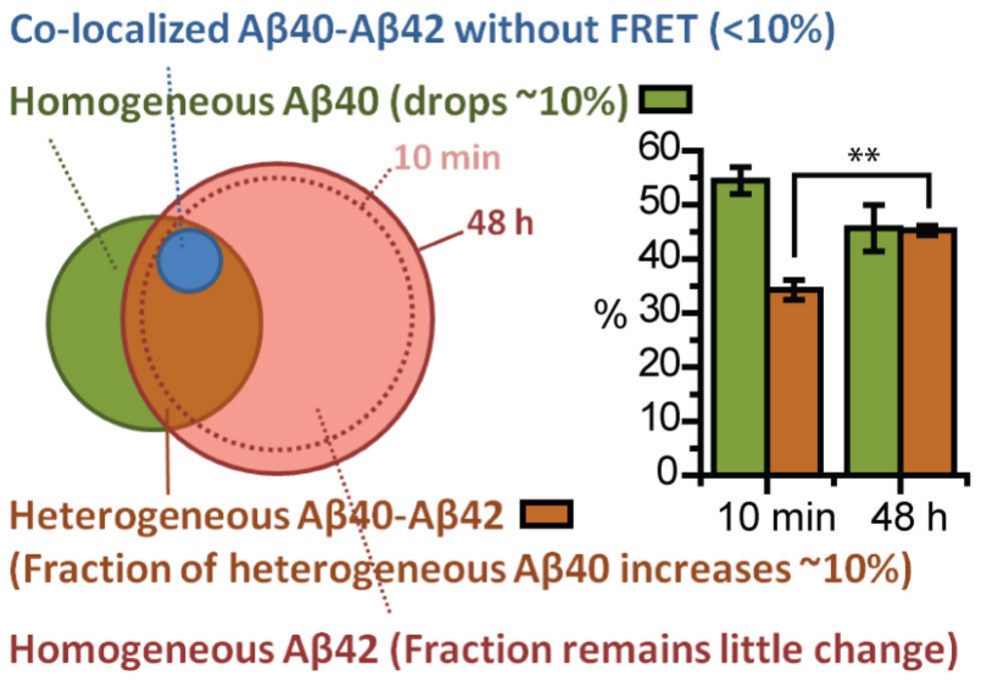
Diagram of the number of Aβ40 and Aβ42 oligomers on the neurites. The color assignments are the same as in Figure 4. Green and red circles represent the total number of Aβ40 and Aβ42 species respectively (including both homogeneous and heterogeneous species). The overlap region of the two circles (brown area) represents the heterogeneous species. And the blue circle inside the brown area represents those heterogeneous species that do not show FRET signal. At 10 minutes, there are slightly more Aβ42 species than Aβ40, therefore larger red circle. By 48 hours, the number of Aβ40 species remains similar; therefore the green circle remains the same. The number of Aβ42 species increases over 48 hours (the dashed red circle depicts the population at 10 minutes). Since additional Aβ42 also binds to homogeneous Aβ40, the fraction of heterogeneous species among Aβ40 increase (unpaired two-tailed t-test, **P < 0.05), and the fraction of homogeneous Aβ40 decreases over 48 hours.

Figure 4A reveals that ~25% of Aβ42 oligomers formed FRET pairs with Aβ40 after 10 minute incubation, and the 25% remained nearly constant over 48 hours. In addition, at 48 hours the total number of Aβ42 oligomers was significantly higher than Aβ40 oligomers (Figure 4B). These results indicate that not only did the additional Aβ42 bind to homogeneous Aβ40 oligomers thereby increasing the number and fraction of heterogeneous Aβ40-Aβ42 over time, but that additional Aβ42 also bound to homogeneous Aβ42 oligomers and likely also formed new oligomers. This explains the increased number of heterogeneous Aβ40-Aβ42 oligomers (and the increased fraction of heterogeneous Aβ40) but the much smaller effect on Aβ42 distribution. This is illustrated by the diagram in Figure 5.

### Heterogeneous Oligomers are Larger than Homogeneous Oligomers

**Figure 6:**
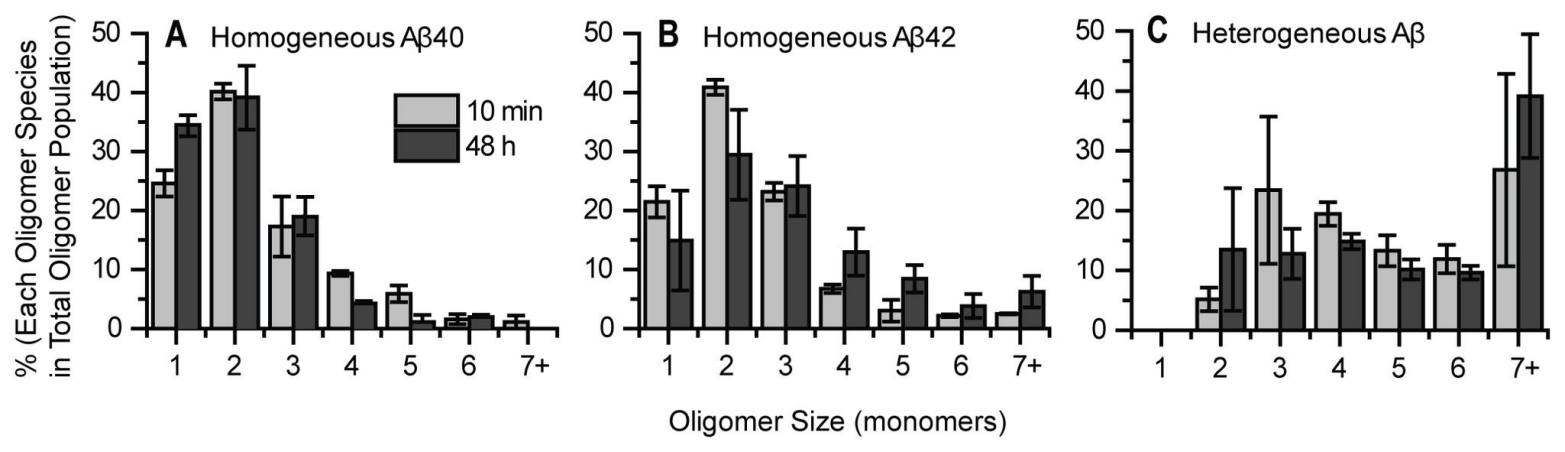
Heterogeneous oligomers are larger than homogeneous oligomers. (A) Homogeneous Aβ40 remains mainly dimeric on the neurites over 48 hours. (B) Homogeneous Aβ42 also forms mainly dimeric with slight increase in size over 48 hours. (C) Heterogeneous species contains mainly trimer and tetramer and many other oligomers larger than heptamer. The size of heterogeneous species was calculated by summing the number of Aβ40 and Aβ42 in that particular mixture. Percentages of each condition were calculated from two different experiments, at least 5 images each. Each image contained at least 50 oligomers. Error bars represent standard deviation of the mean.

In order to study how different Aβ species oligomerizes on the membrane over time, we further analyzed the size of each type of membrane bound oligomer by measuring their fluorescence intensity (see Materials and Methods section). This examination of the relative oligomer sizes (number of peptides in a single oligomer) in the sample containing both Aβ40 and Aβ42 reveals that both homogeneous Aβ40 and Aβ42 remain mostly dimeric over 2 days (Figure 6A and 6B), very similar to samples incubated with only Aβ40 or Aβ42 (Figure 2).

For the heterogeneous oligomers, Aβ40’s emission is quenched due to energy transferring to Aβ42, therefore to obtain the true Aβ40’s original emission intensity, we collected all the photons emitting from both donor and acceptor and corrected the quantum yield and detection efficiency (see Supporting Information). The size of Aβ42 was measured by direct excitation of 635 nm laser. To calculate the size of each heterogeneous oligomer, we rounded each Aβ40 and Aβ42’s calculated size to the nearest integer and summed them up (Figure 6C). The minimal heterogeneous oligomer is of course dimeric (~5%), while ~20% are heptamers or larger at 10 min. This suggests the interactions between Aβ40-Aβ40 and Aβ42-Aβ42 favor dimeric structure on the membrane, where the interaction between Aβ40-Aβ42 favors trimeric, tetrameric and larger structures, which show further growth on the membrane.

### Determining the Relative Fractions of 40 and 42 in Heterogeneous Oligomers

**Figure 7:**
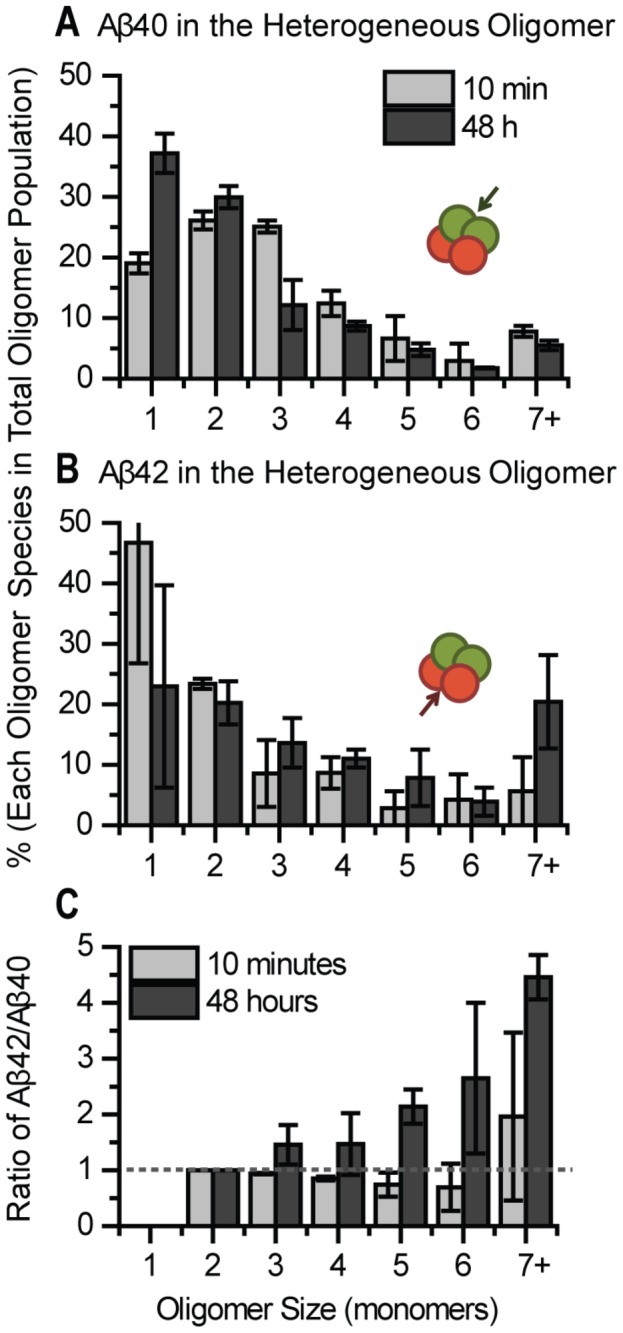
Aβ42 fraction in the heterogeneous oligomers increases dramatically over time but not Aβ40. (A) The size of Aβ40 in the heterogeneous species (as indicated by the green arrow) shifts to smaller species after 48 hours (Mann-Whitney U test, p = 8.6E-7). (B) The size of Aβ42 in the heterogeneous species (as indicated by the red arrow) increases considerably up to 48 hours (Mann-Whitney U test, p = 6.3E-7). (C) The ratio was calculated by dividing the number of Aβ42 monomer by the number of Aβ40 monomer in each individual heterogeneous species. The dashed line indicates ratio 1 at which value the amount of Aβ42 is equal to Aβ40. Data was calculated from two different experiments, at least 5 images each. Each image contained at least 50 oligomers. Error bars represent standard deviation of the mean.

We further analyzed the stoichiometry of Aβ40 and Aβ42 in each heterogeneous oligomer by comparing the size distributions of Aβ40 and Aβ42 inside the oligomer. Results show the fraction of Aβ40 in the heterogeneous species has declined by 48 hours (Figure 7A) which is caused by the continued binding of Aβ42 from solution to homogeneous Aβ40 oligomers (primarily monomeric and dimeric Aβ40) while additional Aβ40 does not bind (Figure 4 and 5). As a result, by 48 hours, the newly formed heterogeneous species contain more monomeric and dimeric Aβ40. Also, the increase in the monomeric fraction is larger than the homogeneous Aβ40, this could indicate that Aβ40 in heterogeneous oligomers may be cleared by cell or dissociate into solution. In contrast, the relative fraction of Aβ42 in the heterogeneous oligomer increased markedly, producing about 4 fold larger oligomers (7+) at 48 hours (Figure 7B). Combining this knowledge with the fact that Aβ42 continues to bind to neurites over time (Figure 4), where the size distribution of homogeneous Aβ42 remains largely constant (Figure 6B), suggests Aβ42 binds equally to homogeneous Aβ42 and new locations on the neurite, but it preferentially binds to the heterogeneous species, increasing the fraction and size of Aβ42 in the heterogeneous species (Figure 7B).

Another way to present the stoichiometric relationship is by calculating the ratio of Aβ42/Aβ40 for each heterogeneous oligomer (Figure 7C). At 10 minutes, there are typically more Aβ40 than Aβ42 in each heterogeneous species (Aβ42/40 ratio < 1, below the dashed line). By 48 hours, there are more Aβ42 adding to the heterogeneous species, shifting the Aβ42/Aβ40 ratio to larger than 1, above the dashed line. This is due to continued binding of Aβ42 from solution to heterogeneous oligomers.

## Discussion

*In vivo* studies have shown that high ratios of Aβ40/Aβ42 may protect neurons from the deleterious effects of Aβ42 [50,51]. This might suggest that lowering the absolute amounts of Aβ in AD patients could be less crucial than the restoration of the correct ratios of Aβ peptides. However, little is known about possible cooperative effects between Aβ42 and Aβ40 under *in vivo* conditions. Aβ42/Aβ40 dependent aggregation kinetics has been measured and extensively studied revealing that a slight increase in the Aβ42 fraction has a significant effect on oligomerization rate and cytotoxicity [20,21,23,52–55]. However, the peptide concentrations used in these studies (µM) were at least 10^3^ times higher than the physiological relevant concentration (pM-nM). Although one could argue the local *in vivo* Aβ concentration might transiently reach µM levels, the overall kinetics and mechanisms of Aβ oligomerization almost certainly behave differently from those at physiological peptide concentrations on neuronal membranes. Moreover, the distribution of the various Aβ oligomers in solution is both concentration and buffer/ionic strength depend, and hence the Aβ oligomers prepared in solution may differ significantly from membrane bound oligomers [56]. To avoid these complications, we directly monitored Aβ40 and Aβ42 oligomers that form on the surface of primary hippocampal neurons under physiological conditions, using single molecule microscopy. Labeling Aβ40 with the FRET donor and Aβ42 with the FRET acceptor reveals the stoichiometry of homogeneous and heterogeneous Aβ oligomers that may further explain the pathogenesis of AD. Direct observation of Aβ on cultured neurons removes the ambiguity caused by SDS-PAGE treatment (See review [56]), and provides structural information for each type of oligomer on or inside the membrane which is new information. 

We have shown that when dissolved as monomeric peptide at nM concentration, both Aβ40 and Aβ42 remain predominantly monomeric (~90%) in solution [38] even after prolonged incubation up to 120 hours (unpublished data). On the cell membrane each peptide formed a distribution of small oligomers peaking at dimers and with less than 10% of the peptide found in species larger than tetramers (Figure 2). Moreover, these oligomers formed quickly (i.e., within 10 min) and showed very little further growth over 48 hours (Figure 2). This behavior has also been reported earlier using single molecule microscopy [36,38,39]. 

Molecular dynamics simulation has shown that Aβ dimerizes strongly when it interacts with anionic lipid membrane [57] which agrees with the findings in our previous work (unpublished data) where Aβ incubated at nM concentration with a model membrane (POPC:POPG 80:20), Aβ initially binds as rapidly diffusing monomers [37] and then slowly oligomerizes to form mostly immobile dimers and trimers. It seems likely that this last step is also the first step for oligomerization of Aβ on the neuronal membrane following binding. 

A plausible explanation for the abundance and immobility of dimers/trimers is that these species incorporate into the membrane (as opposed to be surface bound) and are both more stable and less mobile in this state. Hence the membrane selectively incorporates the dimer/trimer through a direct insertion mechanism [37]. In the case of model membranes the oligomers’ immobilization may indicate that the bound peptide cross the bilayer and become anchored to the surface of the supporting cover slip. A parallel picture for neurites is that binding to surface protein complexes or to intracellular/cytoskeletal elements may be the origin for the oligomers’ immobility.

In the current study we observe that at nM concentration neither Aβ40 nor Aβ42 oligomerizes on the neuronal membrane to form significant populations of oligomers larger than tetramers, even after prolonged incubation. The peptide forms a stable mixture of small oligomeric species that changes very little between 10 min and 48 hours. This behavior is in sharp contrast to Aβ behavior at µM concentrations where the peptide oligomers form rapidly and continuously grow over time to eventually form fibrils within few hours [20,21]. No fibrils appear at 1-4 nM peptide concentrations either in solution or on the neuronal membrane up to 48 hours. Moreover, our previous work revealed that when incubated at nM concentrations with a model membrane, both Aβ40 and Aβ42 develop significantly large oligomers over time (unpublished data); again, this does not happen when the peptides interact with neuronal membranes and is likely due to the equilibrium balance between cell clearance and continued peptide binding from the solution [38].

In what perhaps constitutes the most important observation made in the current study, a dramatic change in the oligomerization reaction sequence was discovered when the cells were exposed to a 1:1 mixture of Aβ40 and Aβ42. Significantly larger membrane-bound oligomers developed within 10 min, with species larger than tetramers constituting over 50% of total peptide (as opposed to less than 10% for homogeneous peptide samples, see Figure 6) and with some additional growth occurring over 48 hours. Concomitantly, the fraction of monomeric peptide completely disappeared and the dimeric fraction was dramatically reduced. 

These observations clearly suggest a synergy of binding between Aβ40 and Aβ42 to the neuronal membrane where initial Aβ40 binding creates “nucleation sites” whose structure favors additional Aβ42 binding to form larger, Aβ42-rich, assemblies. Direct evidence for the formation of these heterogeneous oligomers is provided by FRET (Figures S2, 4, 6, 7 in File S1) which reveals that the chromophores attached to the two different peptides are indeed within several nanometers of each other. An alternate model, where the initial Aβ40 binds to a membrane-associated factor (protein) and this complex binds Aβ42 with high affinity cannot be ruled out; however, there is no compelling indication for this in our data (Aβ40 binding does not show any site-preference).

**Figure 8:**
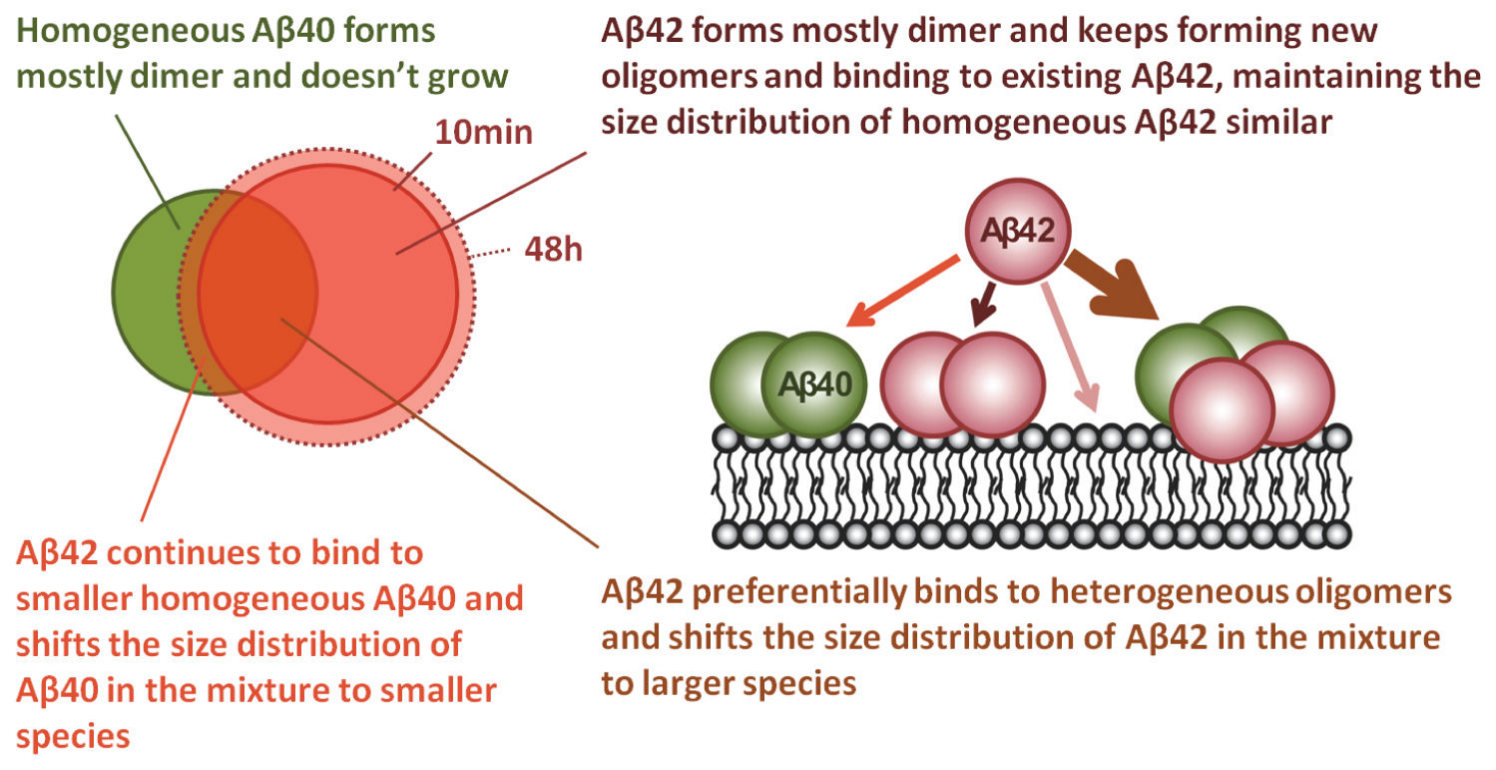
Summary of synergistic interactions between Aβ40 and Aβ42 on the neurons. For Aβ40, the total number of membrane bound oligomers (including both homogeneous and heterogeneous species) does not change, suggesting either no solution Aβ40 binds to the membrane or the association and dissociation of solution Aβ40 to the membrane reach the equilibrium. Size of homogeneous Aβ40 remains mostly dimeric. For Aβ42, the total number of membrane bound oligomers increases. Solution Aβ42 preferentially binds heterogeneous species, increasing the Aβ42/Aβ40 ratio in each mixture. Solution Aβ42 also binds to homogeneous Aβ40, increasing the number of heterogeneous species and shifting the fraction of Aβ40 in the heterogeneous oligomer to a slightly smaller species. However, the solution Aβ42 forms new oligomers and also binds to homogeneous Aβ42, therefore maintaining the size of homogeneous Aβ42 unchanged.

Our results are summarized in Figure 8 and show that when equal concentrations of Aβ42 or Aβ40 were incubated with neurons, slightly more Aβ42 oligomers formed on the neuronal membrane (Figure 4 and 5), and the Aβ42 species grew slightly larger than Aβ40 (Figure 6 and 7). A possible explanation for this is that Aβ42 possesses a higher affinity towards the membrane, hence the higher membrane concentration. In light of recent evidence showing that cells can internalize single Aβ oligomers [58], an alternate explanation is that the clearance of membrane bound Aβ42 is slower than that of Aβ40. Moreover, the oligomers appear to grow exclusively by adding Aβ42 to “seeds” formed by heterogeneous Aβ40/42 and (mostly dimeric) homogeneous Aβ40, which later becomes heterogeneous oligomers and accelerate further attraction of Aβ42, as reflected by the fact that the ratio of Aβ42/Aβ40 increases in individual oligomers with increasing size (Figure 7). 

The importance of this observation is threefold: First, the increase in the size of heterogeneous oligomers may also indicate the oligomer aggregation rate is faster than the cellular clearance rate; second, the presence of membrane bound Aβ40 is necessary for Aβ42 to form heterogeneous oligomers and without Aβ40, the homogenous Aβ42 grows only moderately; and third, the AD brain may contain abundant membrane bound heterogeneous oligomers, which accelerate the association of cerebrospinal fluid (CSF) Aβ42 and increase the burden of Aβ42 in the membrane, resulting in the decreased Aβ42/Aβ40 ratio in CSF but an increased ratio in the plasma membrane. 

A lowering of the level of monomeric Aβ42 in human CSF has been widely validated as a robust biomarker for the diagnosis of AD, even in its earliest clinical stages [41,59–62]. Mechanistically, the progressive accumulation of insoluble Aβ42 enriched deposits in brain parenchyma has been suggested to explain the decline in the level of the highly self-aggregating Aβ42 monomer in both CSF and brain interstitial fluid [41,42]. Aβ42 has been shown to associate with loosely membrane-bound pool of brain parenchyma in plaque rich mice brains, thereby dropping Aβ42/Aβ40 ratio in the CSF but increasing this ratio in the membrane [43]. These observations support the notion that the association of Aβ42 with the membrane is more favorable than the association of Aβ40 and is possibly accelerated by membrane bound heterogeneous oligomers.

Larger Aβ oligomers have been shown to correlate with higher cytotoxicity [18,19]. The finding here that larger peptide oligomers contain increasing fractions of Aβ42 raises the interesting possibility that the higher toxicity is due to the fact that heterogeneous Aβ oligomers are more toxic than homogeneous ones. Jin et al. reported that synthetically made Aβ40 dimers (produced by crosslinking Aβ40 S26C via a disulfide bond) always required much higher concentrations (>100-fold) to induce cytoskeletal disruption comparable to those of the endogenous dimers isolated from AD cortex [63]. Given that heterogeneous oligomers are larger and have been reported in Alzheimer’s disease brain [17], we hypothesize that endogenous Aβ is likely to contain heterogeneous Aβ which form larger oligomers and can cause higher cytotoxicity than synthetic pure Aβ40 or Aβ42. An experiment with cross-linked heterogeneous synthetic dimer could support this hypothesis.

## Conclusion

The oligomeric Aβ species believed to feature in Alzheimer’s disease are known to be numerous and to dynamically interchange, making their characterization challenging and the assignment of disease-related effects to specific oligomers a daunting task. In addition, the concentration of Aβ in bodily fluids is in the nM range or lower, making its study by traditional approaches difficult. Single-molecule microscopy lends itself to work at physiological peptide concentrations and allows one to directly follow the evolution of monomeric Aβ on the neuronal membrane. This is particularly relevant to the detection and characterization of the initial stages of Aβ-induced AD-associated pathology. In the current study, the use of FRET at the single molecule level reveals a strong cooperativity between Aβ40 and Aβ42, where both pure peptides form fewer oligomers larger than dimers on the membrane of cultured neurons, but where membrane bound Aβ40 effectively seeds the addition of Aβ42 to form increasingly larger oligomers. The methodologies employed here may interest other studies in live-cell imaging. Although the rodent and human neuronal membrane may have different composition, the findings here provide detailed insight into structure, dynamics and the mechanism of different types of membrane bound Aβ. Therefore, besides considering the reduction in the quantity of Aβ as a therapeutic strategy, the pathogenic interactions between different Aβ isoforms may also be important. 

## Supporting Information

File S1 **Supporting information.** Figure S1. Sample with Aβ40-HL555 shows shorter fluorescence lifetime spots than the control sample. The raw FLIM data is shown on the left and the calculated lifetime image was fitted with single exponential decay. The fluorescent spots were selected based on the fluorescence image and their lifetimes were collected and plotted as shown on the right. Their lifetime distributions were normalized to total number of spots. The lifetime of Aβ40-HL555 peaks at 0.48 ns and is 6 fold more abundant than the autofluorescence, whereas autofluorescence peaks at 0.58 ns. Therefore we conclude any spot with lifetime longer than 0.53 ns is autofluorescence and excluded. The data presented for each sample is the average of two experiments and each experiment contained at least 250 particles. Error bars represent the standard deviation of the mean. Figure S2. FRET is only detected when Aβ40 is mixed with Aβ42. Primary hippocampal neurons incubated with 2nM Aβ40 were excited by 532 nm laser and show Aβ40 (donor) emission (A) but do not show any emission in Aβ42 (acceptor) channel (B), and Aβ40 can not be directly excited by 635 nm (C). Neurons incubated with 2nM Aβ42 only were also excited by 532 nm laser but do not show any signal in Aβ40 (donor) and Aβ42 (acceptor) emission channels (D and E). The sample with just Aβ42 can only be excited by 635 nm laser and shows emission in Aβ42 (acceptor) emission (F). Neurons incubated with 2nM Aβ40 and 2nM Aβ42 were excited by 532 nm laser and show both donor emission (G) and FRET signal (H). Excitation of 635 nm laser confirmed Aβ42 emission co-localizes with acceptor signals (I). The dashed circle shown in (D) indicates the autofluorescence generated by 532 nm laser, and the donor emission is later distinguished from autofluorescence by their fluorescence lifetime. Scale bars are 10 µm.
(DOC)
