# Supplementary material for: Synergistic Interactions between Alzheimer’s Aβ40 and Aβ42 on the Surface of Primary Neurons Revealed by Single Molecule Microscopy
Source: PLoS One. 2013 Dec 2;8(12):e82139. doi: 10.1371/journal.pone.0082139 (PMC3847093; doi:10.1371/journal.pone.0082139)
Supplement: File S1 — Supporting information. Figure S1. Sample with Aβ40-HL555 shows shorter fluorescence lifetime spots than the control sample. The raw FLIM data is shown on the left and the calculated lifetime image was fitted with single exponential decay. The fluorescent spots were selected based on the fluorescence image and their lifetimes were collected and plotted as shown on the right. Their lifetime distributions were normalized to total number of spots. The lifetime of Aβ40-HL555 peaks at 0.48 ns and is 6 fold more abundant than the autofluorescence, whereas autofluorescence peaks at 0.58 ns. Therefore we conclude any spot with lifetime longer than 0.53 ns is autofluorescence and excluded. The data presented for each sample is the average of two experiments and each experiment contained at least 250 particles. Error bars represent the standard deviation of the mean. Figure S2. FRET is only detected when Aβ40 is mixed with Aβ42. Primary hippocampal neurons incubated with 2nM Aβ40 were excited by 532 nm laser and show Aβ40 (donor) emission (A) but do not show any emission in Aβ42 (acceptor) channel (B), and Aβ40 can not be directly excited by 635 nm (C). Neurons incubated with 2nM Aβ42 only were also excited by 532 nm laser but do not show any signal in Aβ40 (donor) and Aβ42 (acceptor) emission channels (D and E). The sample with just Aβ42 can only be excited by 635 nm laser and shows emission in Aβ42 (acceptor) emission (F). Neurons incubated with 2nM Aβ40 and 2nM Aβ42 were excited by 532 nm laser and show both donor emission (G) and FRET signal (H). Excitation of 635 nm laser confirmed Aβ42 emission co-localizes with acceptor signals (I). The dashed circle shown in (D) indicates the autofluorescence generated by 532 nm laser, and the donor emission is later distinguished from autofluorescence by their fluorescence lifetime. Scale bars are 10 µm. (DOC) [file pone.0082139.s001.doc]

Supporting Information

for

**Synergistic Interactions of Alzheimer’s Aβ40 and Aβ42 on the Surface of Primary Neurons by Single Molecule Microscopy**

Chun-Chieh Chang, John Christian Althaus, Cynthia J. L. Carruthers, Michael A. Sutton, Duncan G. Steel, and Ari Gafni

**Aβ40-HL555 fluorescence is effectively distinguished from cell autofluorescence by time resolved fluorescence measurement.**

A challenge comes from detecting fluorescence emission at single molecules level because cellular autofluorescence competes with emission from exogenous dyes [1,2]. Fluorescence lifetime imaging microscopy (FLIM) has been used to help discriminate this autofluorescence problem [3–5].

While excitation in the red (HL647 or Cy5 dye) results in almost no autofluorescence, an auto-fluorescent signal is generated in the cell when it is excited with a 532 nm laser, which is a common wavelength for the FRET donor. This makes Aβ40-HL555’s emission indistinguishable from the autofluorescence based on the gross emission spectrum (Figure S2D dashed circle). However, incorporating single molecule fluorescence lifetime imaging microscopy (smFLIM) gives us another dimension to differentiate signals with similar fluorescence intensity. We performed smFLIM measurement on two samples, neurons with 2nM unlabeled Aβ40 and neurons with 2nM Aβ40-HL555 (Figure S1 left). The fluorescent spots of these two samples were selected and each spot’s lifetime was fitted to a single exponential decay. For the sample with Aβ40-HL555 we only selected those spots that were away from the cell body which contained strong autofluorescence. The distribution of the lifetime from the selected spots is plotted in Figure S1 right. The lifetime distribution of the control sample peaks around 0.58 ns, whereas the lifetime of membrane bound Aβ40-HL555 peaks around 0.48 ns with 6 fold larger density of emitters than the autofluorescence at this lifetime. Based on this distinct difference, fluorescent spots with lifetime greater than 0.53 ns were considered autofluorescence and excluded from our measurement. While this approach may mark a small number of autofluorescent spots as Aβ40, comparison of the results between two different time points minimizes this bias since the autofluorescence remains similar over time.


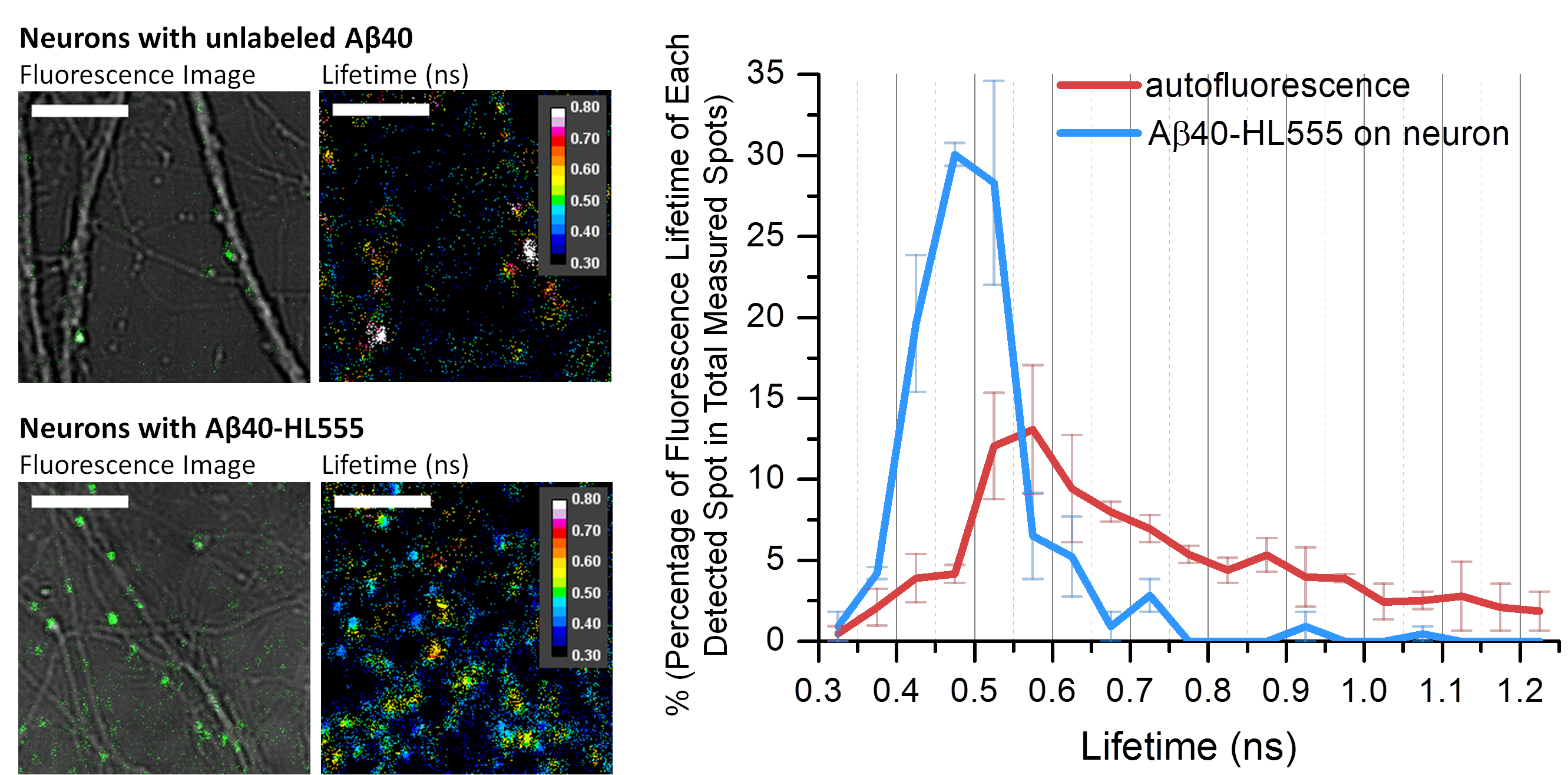


**Figure S1. Sample with Aβ40-HL555 shows shorter fluorescence lifetime spots than the control sample.** The raw FLIM data is shown on the left and the calculated lifetime image was fitted with single exponential decay. The fluorescent spots were selected based on the fluorescence image and their lifetimes were collected and plotted as shown on the right. Their lifetime distributions were normalized to total number of spots. The lifetime of Aβ40-HL555 peaks at 0.48 ns and is 6 fold more abundant than the autofluorescence, whereas autofluorescence peaks at 0.58 ns. Therefore we conclude any spot with lifetime longer than 0.53 ns is autofluorescence and excluded. The data presented for each sample is the average of two experiments and each experiment contained at least 250 particles. Error bars represent the standard deviation of the mean.

**FRET analysis**

When Aβ40 and Aβ42 are mixed together, three different classes of oligomers develop on the neurites: those that contain Aβ40 only (homogeneous oligomer), those made of Aβ42 (homogeneous oligomer), and mixed Aβ40/Aβ42 (heterogeneous oligomer). Only the heterogeneous mixed species show FRET signal (Aβ40 and Aβ42 that are co-localized within the laser beam focus but do not form heterogeneous oligomers do not show FRET). Three quantitative steps are used to identify the FRET species: first, possible FRET pairs are selected by exciting the sample at 532 nm and recording the position of the spot that emitted in Aβ42 channel. Second, those selected fluorescent spots showed in Aβ40 channel with lifetime above 0.53 ns are excluded as autofluorescence. Third, the remaining selected spots showed in Aβ42 channel are double confirmed as the FRET signal by excluding those spots that do not co-localize with spots directly excited by the 635 nm laser. Using this process, the heterogeneous species is filtered out from homogeneous Aβ40 and Aβ42. The homogeneous Aβ40 is determined as those spots that showed fluorescence lifetime below 0.53 ns but do not co-localize with the heterogeneous species. The homogeneous Aβ42 is the one that is directly excited by 635 nm but does not co-localize with the heterogeneous oligomer.

The size of individual Aβ oligomers is quantized based on their relative fluorescence intensity to the monomer intensity. For the homogeneous Aβ40 and Aβ42 as well as for Aβ42 in the heterogeneous species is straightforward because their emission brightness is not significantly modified by quenching. In contrast, when Aβ40 bound to Aβ42, energy transfer occurs and Aβ40 emission is partially quenched. The original intensity of a donor is calculated by

$$I_{A\beta40}^{0}=\frac{I_{A\beta40}}{(1-E)}$$

where
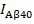
 is the measured Aβ40 emission intensity excited at 532 nm.
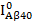
 is the calculated original donor emission before quenching. And E is the FRET efficiency

$$E=\frac{I_{A\beta42}}{I_{A\beta42}+\gamma\times I_{A\beta40}}$$

where
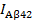
 is the measured Aβ42 emission intensity excited at 532 nm. And γ is the quantum yield of Aβ42 divided by the quantum yield of Aβ40, which has been corrected for its lifetime, and then multiply the detection sensitivity of the acceptor divided by the detection sensitivity of the donor [6].

The degree of bleed through from the donor emission into acceptor channel is calculated by a leaner fit to Aβ40 emission appeared in acceptor channel versus different Aβ40 concentrations in solution containing only the Aβ40. The measured FRET signal is therefore corrected for the bleed through from its donor (Aβ40). The direct excitation of the acceptor at 532 nm was found to be insignificant.

We note that in using the FRET efficiency we estimate the distance between two N-terminus of a dimer is ~60Å. This is larger than would be expected for a globular association of the peptides forming the oligomer. There are at least three possibilities that might account for this. The first is that the assembly is not globular but stretched out, perhaps because it is on a surface, though this seems unlikely. The second is that the calculation for the FRET distance assumes that the dipole orientation between the donor and the acceptor is fully randomized (i.e., k=2/3). If assembly on the lipid bilayer results in steric hindrance, then k could be much smaller resulting in lower transfer efficiency and an increased inferred separation between donor and acceptor. A third possibility is that these oligomers are not structures of pure Aβ peptides, but rather structures that assemble on the membrane surface either by association with some structural scaffold (e.g., a membrane bound protein complex or something associated with the cytoskeletal structure) or by including lipids in the structure leading to a larger assembly. Resolving these models by optical imaging clearly lies outside the bound of even the highest super resolution methods. Regardless of the microscopic picture that ultimately explains the FRET efficiency, the conclusion from the data remains sound, namely that these structures grow only by the original Aβ40 assembly recruiting Aβ42. And the molecular description of the structure of the self assembly is secondary to the potential importance of this effect in the development of cellular toxicity.


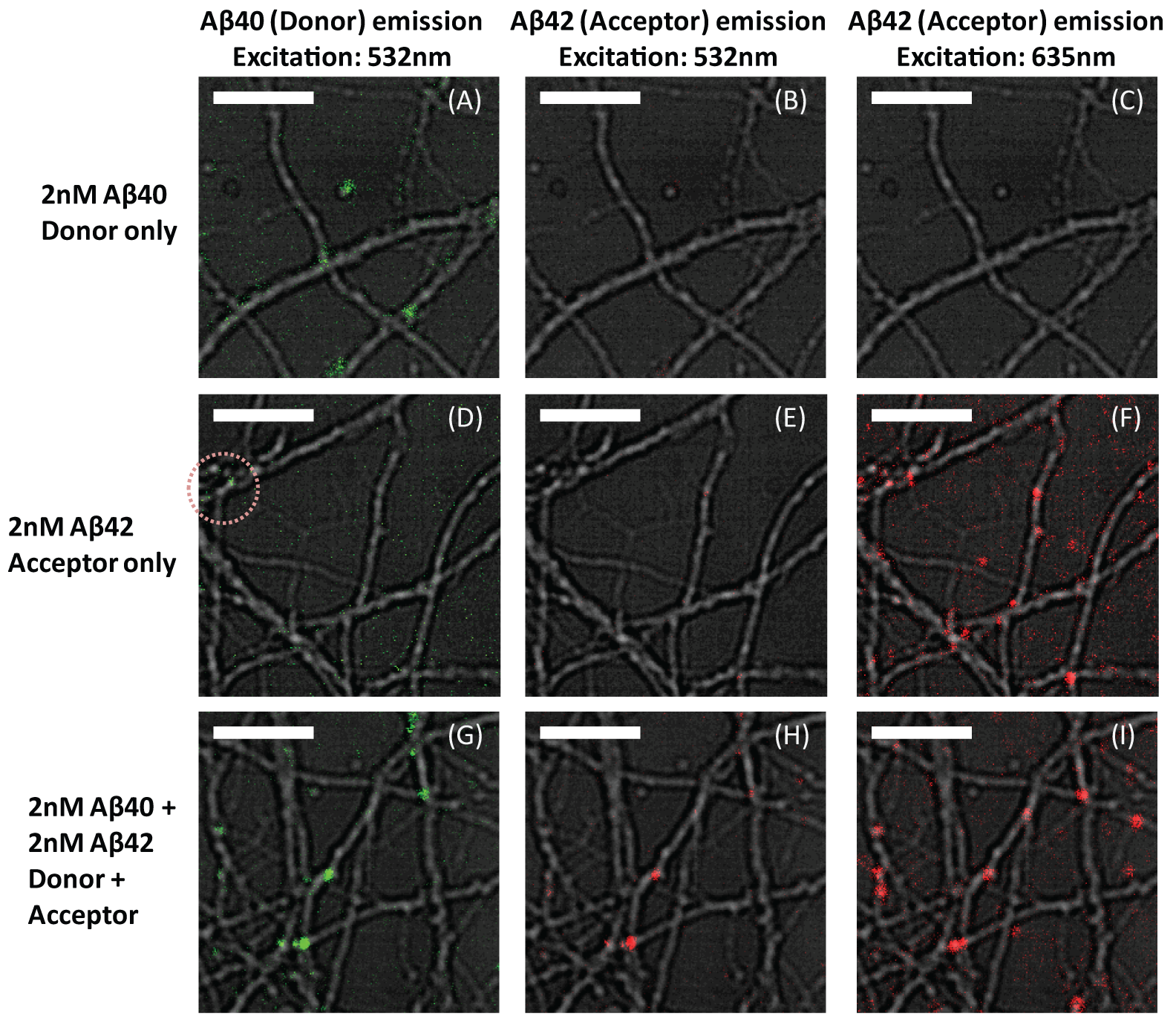


**Figure S2. FRET is only detected when Aβ40 is mixed with Aβ42.** Primary hippocampal neurons incubated with 2nM Aβ40 were excited by 532 nm laser and show Aβ40 (donor) emission (A) but do not show any emission in Aβ42 (acceptor) channel (B), and Aβ40 can not be directly excited by 635 nm (C). Neurons incubated with 2nM Aβ42 only were also excited by 532 nm laser but do not show any signal in Aβ40 (donor) and Aβ42 (acceptor) emission channels (D and E). The sample with just Aβ42 can only be excited by 635 nm laser and shows emission in Aβ42 (acceptor) emission (F). Neurons incubated with 2nM Aβ40 and 2nM Aβ42 were excited by 532 nm laser and show both donor emission (G) and FRET signal (H). Excitation of 635 nm laser confirmed Aβ42 emission co-localizes with acceptor signals (I). The dashed circle shown in (D) indicates the autofluorescence generated by 532 nm laser, and the donor emission is later distinguished from autofluorescence by their fluorescence lifetime. Scale bars are 10 µm.

**REFERENCES**

1. Benson RC, Meyer RA, Zaruba ME, McKhann GM (1979) Cellular autofluorescence--is it due to flavins? The journal of histochemistry and cytochemistry : official journal of the Histochemistry Society 27: 44–48.

2. Aubin JE (1979) Autofluorescence of viable cultured mammalian cells. The journal of histochemistry and cytochemistry : official journal of the Histochemistry Society 27: 36–43.

3. Lundin K, Blomberg K, Nordström T, Lindqvist C (2001) Development of a time-resolved fluorescence resonance energy transfer assay (cell TR-FRET) for protein detection on intact cells. Analytical biochemistry 299: 92–97.

4. Zhou V, Han S, Brinker A, Klock H, Caldwell J, et al. (2004) A time-resolved fluorescence resonance energy transfer-based HTS assay and a surface plasmon resonance-based binding assay for heat shock protein 90 inhibitors. Analytical biochemistry 331: 349–357.

5. Xu Z, Nagashima K, Sun D, Rush T, Northrup A, et al. (2009) Development of high-throughput TR-FRET and AlphaScreen assays for identification of potent inhibitors of PDK1. Journal of biomolecular screening 14: 1257–1262.

6. Roy R, Hohng S, Ha T (2008) A practical guide to single-molecule FRET. Nature methods 5: 507–516.
